# The Texas Special Committee in Surgery

**Published:** 1887-11

**Authors:** 


### "THE TEXAS SPECIAL COMMITTEE ON SURGERY."

Under this caption Dr. Davis devotes over two pages (four columns), in the *Journal of the American Medical Association*, of November 5th, to a review of the work compiled by our special Committee on Surgery, and published as a supplement to the State Medical Association transactions. He enters iuto a minute examination and criticism of the several tables, giving, in detail, the several summaries.

This shows that the eyes of the profession of the world are not only on the Texas medical profession, but that their work is critically examined and compared with the work of other States, and is found full of interest. The fact that this distinguished editor of the official organ of the American Medical Association should make this work the subject of the *leading editorial*, is a high compliment —even if his criticism had been adverse; but when he speaks of that work in terms of the highest recommendation, and holds it up to the profession as an example, worthy of emulation, it should make every member of the Texas Medical Association feel proud. We feel an especial pride in the matter, and rejoice to see the labors of our devoted Cupples—the crowning glory of his usefûl life, so well appreciated—for, Dr. Cupples though too modest to claim it, is entitled to the credit of this laborious compilation, and the beautiful manner in which it was brought out by the San Antonio printers,—aside from the pride that we all feel in seeing the work so favorably compared with that of other societies. Dr. Davis says snch reports are of great value, and show that the Surgeons of Texas recognize and do their duty to the profession, by contributing to the stock of common knowledge; that the Texas State Medical Association is fully alive to the interests of the profession in appointing such a committee; and that "State Medical Association can find a committee to do good work."

We hope that this will stimulate our surgeons to come forward more promptly and give Dr. Cupples the notes of their cases for the next report; for we know that some have questioned the value of such publications and have held back, and refused to contribute to the work. We know also that there are some who now express regret that they did not comply with the polite invitation sent them by the committee, and report their cases for the present volume.

